# The effect of meteorological factors on airborne *Betula* pollen concentrations in Lublin (Poland)

**DOI:** 10.1007/s10453-012-9249-z

**Published:** 2012-01-31

**Authors:** Krystyna Piotrowska, Agnieszka Kubik-Komar

**Affiliations:** 1 Department of Botany, University of Life Sciences in Lublin, Akademicka 15, 20-950 Lublin, Poland; 2 Department of Applied Mathematics and Computer Science, University of Life Sciences in Lublin, Akademicka 13, 20-950 Lublin, Poland

**Keywords:** *Betula*, Atmospheric pollen season, Meteorological parameters, Regression analysis

## Abstract

The present study investigated the pattern of the birch atmospheric pollen seasons in Lublin in the period 2001–2010. Pollen monitoring was conducted using a Lanzoni VPPS 2000 sampler. The atmospheric pollen seasons were determined with the 98% method. Regression analysis was used to determine correlations between meteorological conditions and the pattern of the birch pollen season. On average, the birch pollen season started on 12 April, ended on 13 May, and lasted 32 days. The peak value and the Seasonal Pollen Index showed the greatest variation in particular years. All the seasons were right-skewed. During the study years, a trend was found towards earlier occurrence of the seasonal peak. Regression equations were developed for the following parameters of the atmospheric pollen season: start, duration, peak value and average pollen concentration during the season. The obtained model fit was at a level of 64–81%. Statistical analysis shows that minimum temperature of February and March and total rainfall in June in the year preceding pollen release have the greatest effect on the birch atmospheric pollen season in Lublin. Low temperatures in February promote the occurrence of high pollen concentrations.

## Introduction

Recent research shows that in Poland over 45% of its population (that is, more than 15 million residents) suffer from various allergies. These diseases are primarily prevalent among children and young people (Samoliński et al. 2007). In spring, birch pollen grains are the main cause of pollen allergy in northern and central Europe. It is estimated that 10–20% of the population in this region are allergic to birch pollen (Spieksma 1990). Allergy to birch pollen is usually accompanied by allergy to alder and hazel pollen grains. Besides, cross-reactions are observed between birch pollen allergens and some fruits. Hypersensitivity to apples, pears and peaches has been found in approximately 30% of patients allergic to birch pollen (Rapiejko 2005). Allergy symptoms appear suddenly in people allergic to birch pollen, without initial symptoms gradually developing as it is in the case of allergies to other allergens (Rapiejko 2005). This is associated with very high concentrations of birch pollen grains at the beginning of the season. Pollen shed from anthers lasts on average 10 days for *Betula pendula* and 15 days for *Betula pubescens.* However, most pollen, that is, 70–80%, is shed within 2–3 days (Suszka 1979).

Birch produces huge amounts of pollen grains. It has been calculated that one male flower of *Betula pendula* produces ca. 22,000 pollen grains, while one inflorescence bearing 450 flowers produces more than 10 million grains (Piotrowska 2008). In Poland, birch belongs to the tree plant taxa that reach the highest annual totals and daily pollen concentrations in the air (Weryszko-Chmielewska 2006). The period of time during which pollen is present in the atmosphere is named atmospheric pollen season (Jato et al. 2006). Given the increase in the number of people allergic to plant pollen, special attention is paid to predicting the start dates and severity of atmospheric pollen seasons of allergenic plants. It is very important to predict when and how abundantly trees will release pollen.

Likewise, in the case of other plants blooming in early spring, the onset of pollen shed in birch is closely related to weather conditions and primarily depends on air temperature (Emberlin et al. 1997; Norris-Hill 1998; Stach et al. 2008a). The number of pollen grains in a particular pollen season is also determined by the conditions prevailing in the previous year, during the formation of flower buds (Latałowa et al. 2002).

The timing and intensity of birch pollen seasons vary significantly in particular years. The start date of the pollen season is most frequently predicted based on regression analysis (Adams-Groom et al. 2002; Rodriguez-Rajo et al. 2003). We are convinced that it is also important to predict season duration and severity. The aim of the present study was to analyse the variation in birch atmospheric pollen seasons in particular years and to determine which meteorological factors had the greatest effect on the features of the *Betula* pollen season in Lublin. On this basis, an attempt was made to develop models that would show the correlations between the parameters of the atmospheric pollen season and meteorological factors. These models are the first step towards predicting the timing and abundance of the birch pollen season in Lublin. The possibility of predicting beforehand the season start date and severity would be a valuable help for both allergists and allergic people. In the first part of paper, weather conditions during the atmospheric pollen seasons using PCA were analysed. Next, pollen seasons were compared based on pollen data from 10-year monitoring (cluster analysis). At last, the relations between pollen seasons parameters and meteorological data by means of regression analysis were searched.

## Materials and methods

### Aerobiological analysis

Pollen monitoring was performed in Lublin (eastern Poland) in the period 2001–2010. The climate of the Lublin region is characterized by the influence of continental air masses. The general growing season lasts 215 days there (Woś 1999). Mean annual air temperature in Lublin (1951–2007) is 8.2°C, and January is the coldest month of the year (mean temperature −2.8°C). In February, average temperature reaches −1.9°C, whereas in March and April it is 2.0 and 8.5°C, respectively. Mean annual total precipitation in Lublin is 546 mm (Kaszewski 2008; Piotrowska and Kaszewski 2009). The genus *Betula* is represented in the region of Lublin by the following species: *B. pendula, B. pubescens* and *B. humilis* (Zając and Zając 2001). The taxon chosen for study is very common in Lublin and its vicinity. We have observed that birch tree is most abundant in all districts of the city.

The sampling site was located close to the city centre (λ = 22°32′25″E and φ = 51°14′37″N; 197 m a.s.l.). A Hirst-type sampler (Lanzoni VPPS 2000) was used for pollen trapping. It was placed on the flat roof of a building of the Lublin University of Sciences at a height of 18 m. Pollen grains were identified in 4 horizontal traverses of the slide. The results were expressed as the number of pollen grains per 1 m^3^ of air in 24 h (P/m^3^; Mandrioli et al. 1998).

The following atmospheric pollen season (APS) parameters were analysed: start, end, duration, maximum pollen concentration (peak value), date of maximum concentration and Seasonal Pollen Index (SPI) (the summation of the daily mean concentration during the pollen season Mandrioli 1998). Two methods 98% (Emberlin et al. 1993) and 95% (Andersen 1991) were considered to define the atmospheric pollen season. The 98% method was used to determine the atmospheric pollen season (Emberlin et al. 1993; Emberlin et al. 2002). The start of the season was defined as the date when 1% of the seasonal cumulative pollen count was trapped and the end of the season when the cumulative pollen count reached 99%. The use of 95% method would have removed too much data from the analysis.

### Meteorological data

The meteorological data came from the Meteorological Observatory of the Meteorology and Climatology Department, the Maria Curie-Sklodowska University in Lublin; the Observatory is located at a distance of about 1.5 km from the pollen sampling site. The following meteorological data were used for the analysis: air temperature (mean, minimum and maximum), relative air humidity, rainfall, cloud cover and wind speed. In the PCA, daily weather conditions during pollen seasons were taken into account. Monthly meteorological data starting from May in the year preceding pollen release until May in the year of pollen emission, average meteorological data from the pollen seasons (Latałowa et al. 2002; Smith et al. 2009), average daily meteorological conditions for the periods of 10, 20, 30, 40 and 50 days before the atmospheric pollen season start date as well as start and cumulative air temperature (>0 and >5°C) were variables, influence of which on pollen features was investigated. Not all above-mentioned data were included into regression analysis—the dataset was limited on the basis of Spearman’s correlation results.

### Statistical analysis

Classical statistical parameters, such as arithmetic mean, minimum and maximum values, standard deviation (SD) and coefficient of variation (V), were used to characterize the features of the birch atmospheric pollen seasons. In order to determine the direction and strength of skewness, the coefficient of skewness was calculated; it has a zero value when the distribution of the variable is symmetric. A positive value of this coefficient indicates a right skew, while a negative value means a left skew (Wołek 2006).

In addition to descriptive statistics, some uni- and multivariate statistical methods were applied to determine the effect of meteorological conditions on the pattern of the birch atmospheric pollen season. Principal component analysis (PCA) was used to compare weather conditions during the pollen seasons (Lighthart et al. 2009). Daily values of mean temperature, minimum temperature, rainfall, humidity, cloud cover and wind speed during *Betula* pollen season were the studied variables. Maximum temperature was excluded from the analysis because of the very high correlation with mean temperature (0.97). The results are presented in the form of a table of factor loadings showing correlations between weather parameters and the obtained factors (PC1, PC2 and PC3) as well as in the form of factor score scatterplots with the PC1-PC2, PC1-PC3 and PC2-PC3 coordinate systems (González Parrado et al. 2009). Factor loadings were obtained after VARIMAX rotation maximizing the sum of the variances of the squared loadings (Ferguson and Takane 1989).

The differences among atmospheric pollen seasons were compared by applying cluster analysis. The daily pollen concentration during 2001–2010 years was treated as dataset for this analysis. These results were presented as a hierarchical tree plot where the distance of linkage is a measure of similarity of pollen seasons. The joining algorithm was based on the Euclidean distance and the centroid method of linkage (Romesburg 2004). An example of cluster analysis application in other studies involving comparison of pollen seasons can be found in the paper of Latorre and Belmonte (2004). In most articles on cluster analysis, however, the method is used to identify clusters of sites with similar pollen concentration profiles (Orlandi et al. 2005; Rieux et al. 2008; Smith et al. 2009). The above-described methods of multivariate statistical analyses were applied to characterize each of the pollen seasons compared with other seasons (according to weather conditions or pollen concentration). 

Regression analysis was applied to describe the relationships between weather and pollen season parameters. The univariate and multivariate versions of this method (Ferguson and Takane 1989) were used, where the season start date, season duration, peak and mean pollen concentration were dependent variables and monthly mean values of weather parameters, selected on the basis of Spearman correlation values, were independent ones (Stach et al. 2008b; Frei and Gassner 2008; Smith et al. 2009).

All statistical calculations were performed using STATISTICA and SPSS software.

## Results

### Meteorological data during the atmospheric pollen seasons: principal component analysis (PCA)

Before the analysis was performed, the data were standardized in order to eliminate differences in the investigated parameters resulting from the differences in measurement units. The analysis results are presented in Table 1 that shows factor loadings after VARIMAX rotation, that is, the correlation coefficients between weather factors and the derived three components—PC1, PC2 and PC3. Because PC1 was most strongly correlated to rainfall and cloud cover, this factor was termed as inclement weather. The values of the factor loading PC2 showed that this factor was influenced to the greatest degree by temperature; therefore, it was determined in this way. The last factor, PC3, was negatively correlated with wind speed; hence, we interpret it as windless weather.

**Table 1:** Factor loadings after VARIMAX rotation

Weather parameters	PC1	PC2	PC3
Mean temperature	−0.23	0.95*	0.06
Minimum temperature	0.20	0.95*	−0.07
Rainfall	0.71*	0.09	0.07
Humidity	0.89*	−0.11	0.03
Cloudiness	0.82*	−0.02	−0.11
Wind speed	−0.01	0.01	−0.99*

* Factor loading value >0.70

**Fig. 1:**
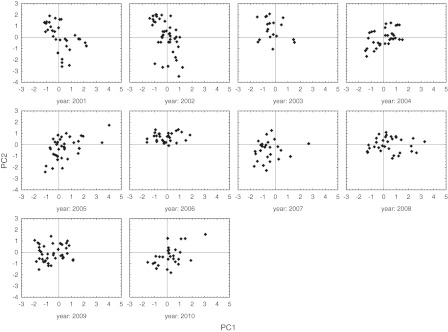
Factor scores of seasons 2001–2010 in PC1-PC2 coordinate system

The determined factors explain 81.7% of the variability of the system under consideration. The observation vectors, consisting of the values of the individual meteorological factors, are shown in the coordinates PC1, PC2 and PC3 using factor scores. The scatter plots for these values in the coordinates PC1-PC2, with a breakdown into consecutive seasons, are shown in Fig. 1, and they present large variations in particular seasons due to inclement weather and temperature. In terms of temperature, the atmospheric pollen seasons in 2001 and 2002, but also in 2005, exhibited the greatest variation, whereas in 2008, season was characterized by the highest weather variability. Moreover, it can be seen that in 2003 and 2006 atmospheric pollen seasons, nearly all observations are above the PC1 axis, which means that these seasons can be considered to be warm, contrary to seasons in 2010 and 2007. The scatter plots for the systems of PC1-PC3 and PC2-PC3 (available on request) show that the diversity of factor scores alongside the PC3 coordinate is very high in each case, which means that there were both windy and calm days observed in all seasons.

**Fig. 2:**
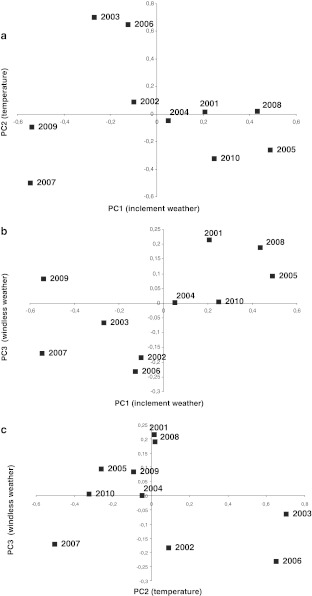
Mean value of factor scores for 2001–2010 seasons

In order to compare weather conditions during the atmospheric pollen seasons, the mean factor scores were calculated for each season, and these values are shown in Fig. 2. Pollen seasons in 2003 and 2006 were the warmest, whereas in 2007 was the coldest one (Fig. 2a, c). Atmospheric pollen seasons were characterized in 2007 and 2009 by fine weather, while cloudiness, humidity and rainfall could most often be observed in the seasons in 2005, 2008 and 2010 (Fig. 2a, b). Strong wind was observed in the pollen seasons in 2002, 2006 and 2007, while in 2001 and 2008 wind speed was the lowest (Fig. 2b, c).

As the last step of this analysis, the Spearman’s correlations between obtained PCA factors and pollen concentration were calculated. The values of correlation were very low (−0.164, 0.049 and 0.021) what means that *Betula* pollen concentration was influenced by meteorological data during the atmospheric pollen seasons to a small extent. This is why other metrological factors were taken into consideration for regression analyses.

### Aerobiological data

#### Start and end of atmospheric pollen season

The birch atmospheric pollen season in Lublin started on average on 12 April (102nd day of the year) and ended on 13 May (day 133; Table 2). Its start was recorded earliest in 2002, after exceptionally warm February and March. At that time, mean air temperature was higher than the long-term average for 2001–2010 by 4.1 and 2.1°C, respectively. The latest start of APS (2006) was preceded by a period of low temperatures. In 2006, as the only year during the period 2001–2010, average temperature of January, February and March maintained below 0°C. The pollen season start date was characterized by greater variation than the end of its date (Table 2).

**Table 2:** Statistics of the parameters of the birch pollen season in Lublin in 2001–2010

Statistics	Pollen season			Peak		SPI
	Start	End	Duration (days)	P/m^3^	Date	
Mean	12.04	13.05	32	3,574	19.04	16,146
Min	3.04 (2002)	3.05 (2010)	19 (2003)	521 (2009)	13.04 (2010)	3,219 (2009)
Max	22.04 (2006)	21.05 (2005)	42 (2002)	12,832 (2003)	26.04 (2001)	33,788 (2003)
SD	6.1	5.8	6.7	3,524.4	4.6	9,719.3
*V* (%)	6.0	4.3	21.1	98.6	4.2	60.2

#### Duration of atmospheric pollen season

On average, the birch atmospheric pollen season lasted 1 month; it was the shortest in 2003 (19 days) and longest in 2002 (42 days). During the pollen season, the most favourable thermal conditions were recorded in 2003 when mean air temperature was higher than the average for the 10-year study period by 2.7°C. The lowest precipitation total was then recorded (lower by 26.9 mm than the long-term average for 2001–2010). During 2002, all the meteorological factors were close to the means for the 10-year study period.

#### Peak and SPI

The highest pollen concentrations in all the study years were observed in the second half of April. In 2003, the atmospheric pollen season had the most intense pattern. In this year, the value of maximum pollen concentration was 25 times higher than in the year 2009 in which the pollen concentration was the lowest. A statistically significant negative correlation was found between the APS duration and maximum pollen concentration as well as the SPI. The maximum values and the SPI were very strongly positively correlated (Table 3).

**Table 3:** The list of significant Spearman’s correlations between the parameters of the birch pollen season in Lublin (2001–2010)

Parameters of pollen season	Spearman coefficient
Duration & peak value	−0.689037*
Duration & SPI	−0.762209*
Peak value & SPI	0.975758**
Days number of pre-peak & season start	−0.666670*
Days number of post-peak & season end	0.693304*
Days number of post-peak & duration	0.695135*

Level of significance * 0.05; ** 0.01

Among the parameters of the APS, the highest variation was noted for the peak value (*V* = 98.6%). But the date of the seasonal maximum was the most stable parameter of the APS that was characterized by the lowest variation (Table 2).

**Fig. 3:**
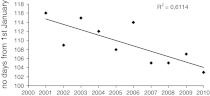
Occurrence dates of the seasonal maximum in Lublin in 2001**–**2010

During the study years, a distinct trend towards earlier occurrence of the peak value for birch pollen concentration was observed (Fig. 3).

**Fig. 4:**
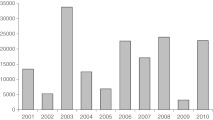
Seasonal Pollen Index (SPI) for the birch pollen seasons in Lublin in 2001–2010

Pollen counts observed in the APS in Lublin significantly varied in particular years. The lowest numbers of pollen grains were recorded in 2002 and 2009, while the highest ones in 2003. Generally, lower annual pollen counts were recorded after a year with a high pollen count, but no clear biennial pattern of high pollen concentrations was found (Fig. 4).

#### The pattern of atmospheric pollen seasons

A negative correlation was found between the number of days in the pre-peak period and the pollen season start date; the later the season started, the earlier the peak occurred. The number of days from the peak date until the end of the season was higher when the latter lasted longer and ended later (Table 3).

The pattern of the atmospheric pollen seasons in Lublin was right-skewed. A rapid increase in pollen concentration was noted, and subsequently a much slower decrease. In all the study years, the coefficient of skewness had a positive value. The highest skewness value was recorded in the years 2005 (3.51) and 2003 (3.18), whereas the lowest one in 2004 (0.96). In the other years, the values of the skewness coefficient were in the range of 1.52–2.43. The average number of days in the pre-peak period was much lower than in the post-peak period. The highest pollen concentration was recorded during the first days of the APS, on average after 7 days from the season start date. The number of days from the peak date until the end of the season was 24 days, on average.

#### The cluster analysis

**Fig. 5:**
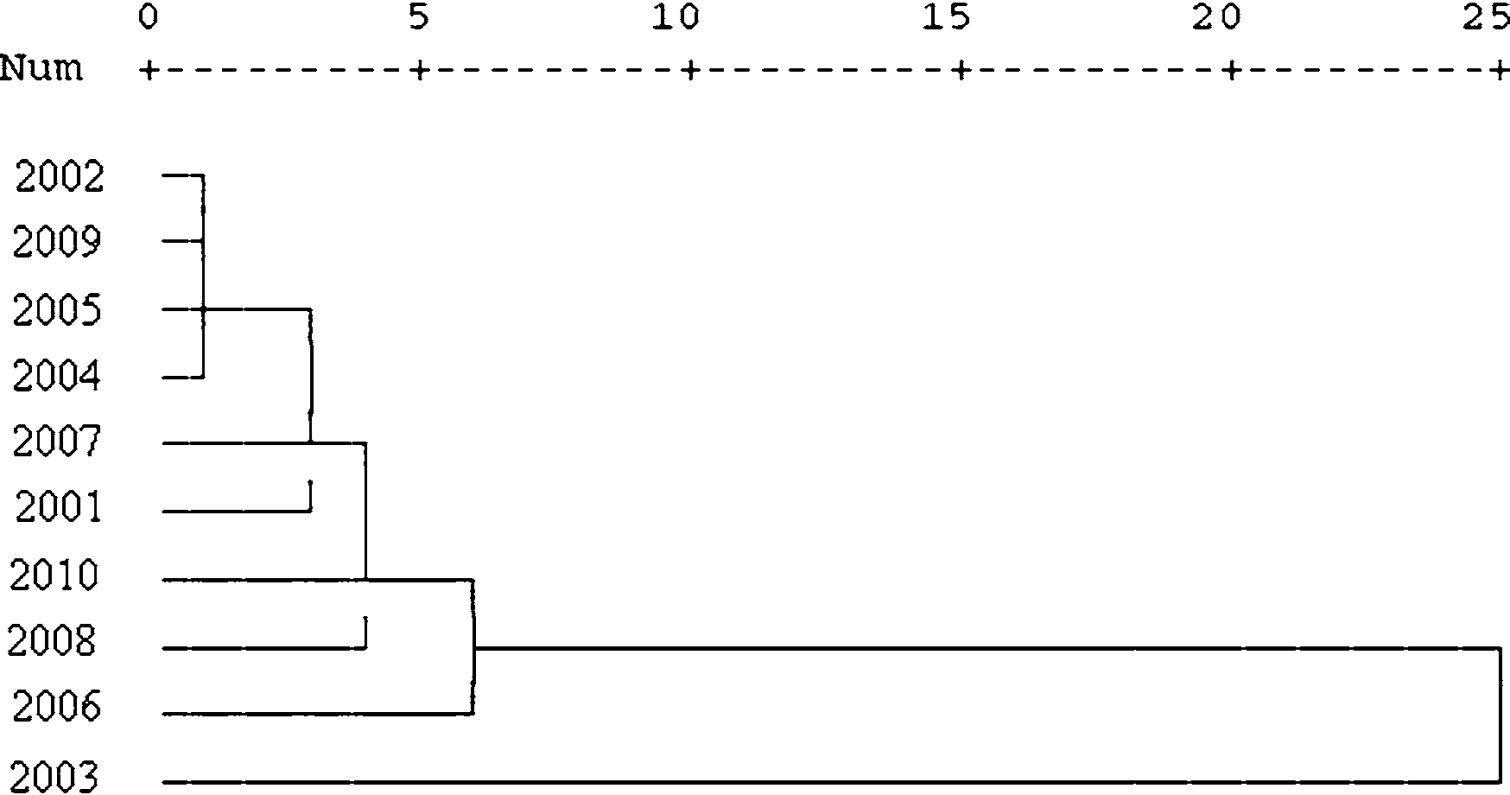
Cluster analysis for 2001–2010 pollen seasons

The cluster analysis results are shown in a dendrogram (Fig. 5). The seasonal dynamics of airborne birch pollen concentration in Lublin showed high variability. The atmospheric pollen seasons in the years 2002, 2004, 2005 and 2009 were the most similar and were characterized by early start, low pollen concentrations and close patterns. Comparable pollen count and pattern of APS were observed in 2006, 2008 and 2010. In 2003, APS appeared to be totally different (Fig. 5). It was probably caused by the late start of the pollen season and very high pollen concentration compared with other seasons.

### Regression analysis

Due to short data series, all the study years were included to obtain a better fit to the regression model. When creating the regression model, the number of independent variables was reduced on the basis of Spearman’s correlation coefficients test, and only the statistically significant ones were used. The selection of the best-fitted model was based on the Adj *R* ^2^ value. A relatively good fit was obtained for the start date and duration of the atmospheric pollen season as well as for the value of maximum and average concentrations during the atmospheric pollen season.

**Fig. 6:**
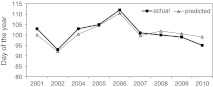
The start dates of the birch pollen season; actual versus regression model predicted values

Minimum temperature in February was the factor that was most correlated with the atmospheric pollen season start date. No statistically significant correlations were found between the season start and average meteorological conditions as well as cumulative temperatures in the periods of 10, 20, 30, 40 and 50 days before the season. As a result of regression analysis, an adjusted determination coefficient was obtained, equal to Adj *R* ^2^ = 0.73. Thus, the derived model explains about 73% of the variation in the start dates of the birch atmospheric pollen seasons. A better fit to the regression model was obtained after the extreme year 2003 had been excluded (Fig. 6).

$$ \begin{gathered} {\text{PS}} = 102.42 - 3.36*T_{{\min
({\text{II}})}} - 2.25*T_{{\min ({\text{II}})}}^{2} -
0.30*T_{{\min ({\text{II}})}}^{3} \hfill \\ {\text{Adj}}\,R^{2} =
0.732 \hfill \\ \hfill \\ \end{gathered} $$

PS, start atmospheric pollen season; *T*_min(II)_, mean minimum temperature in February.

**Fig. 7:**
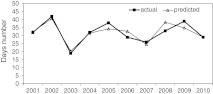
The duration of the birch pollen season; actual versus regression model predicted values

The duration of the birch APS was primarily dependent on minimum temperature in February and March. The difference between the regression model predicted values and the actual values was on average 2 days (Fig. 7).

$$ \begin{gathered} {\text{DS}} = 40.69 + 2.52T_{{\min ({\text{II}})}} - 2.45T_{{\min ({\text{III}})}} \hfill \\ {\text{Adj}}\,R^{2} = 0.73  \hfill \\ \hfill \\ \end{gathered} $$

DS, duration of atmospheric pollen season; *T*_min(II)_, mean minimum temperature in February; *T*_min(III)_, mean minimum temperature in March.

**Fig. 8:**
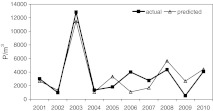
The peak of the birch pollen season; actual versus regression model predicted values

In order to find a relationship between pollen concentration and weather, the meteorological data from the previous year and from the year of pollen release were analysed. Rainfall in June of the year preceding pollen release, that is, during the time when male flowers develop, was found to have the greatest effect on the peak concentration of birch pollen. This variable (*R*_VI(*Y*−1)_) explains the variation in the maximum values of birch pollen concentrations in ca. 81% (Fig. 8).

$$ \begin{gathered} {\text{PV}} = 17062.8 - 946.37*R_{{{\text{VI}}(Y - 1)}} + 16.81*R_{{{\text{VI}}(Y - 1)}}^{2} - 0.078*R_{{{\text{VI}}(Y - 1)}}^{3} \hfill \\ {\text{Adj}}\,R^{2} = 0.808  \hfill \\ \hfill \\ \end{gathered} $$

PV, peak value; *R*_VI(*Y*−1)_, rainfall in June of the year preceding pollen release.

**Fig. 9:**
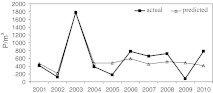
Mean *Betula* pollen count in pollen season; actual versus regression model predicted values

The best fit for the SPI was sought. No satisfactory result was achieved. A better fit was obtained when the average birch pollen concentration during the season was taken into account (Fig. 9). The correlation of this trait with meteorological factors is described by the below equation:

$$ \begin{gathered} y = 532.59-109.03*T_{{\min ({\text{II}})}} -65.47*T_{{\min ({\text{II}})}}^{2} -7.81*T_{{\min ({\text{II}})}}^{3} \hfill \\ {\text{Adj}}\,R^{2} = 0.64  \hfill \\ \hfill \\ \end{gathered} $$

*y*, mean birch pollen count during atmospheric pollen season; *T*_min (II)_, mean minimum temperature in February.

## Discussion

Regression analysis is used by many authors to predict and determine the effect of weather on pollen seasons (among others, Emberlin et al. 1993; Dahl and Strandhede 1996; Adams-Groom et al. 2002; Latałowa et al. 2002; Rodriguez-Rajo et al. 2003). Meteorological conditions are the main factors that modify pollen seasons, but they are not the only ones; this explains the fact why it is so difficult to find a very good fit of a predictive model. The time of pollen shed in birch is dependent, *inter alia*, on the soil on which birch trees grow. Birch trees growing in peat and wetland shed pollen later and longer than trees growing on compact soils (Suszka 1979). Endogenous resources are also of great importance; in the biology of tree growth, the development of a large leaf assimilation area and, subsequently, development of a large number of inflorescences occur alternately in successive years (Dahl and Strandhede 1996). The production of a large number of pollen grains in 1 year leads to abundant fruiting, which entails a high energy expense and results in inhibition of the growth of inflorescences in the next year (Suszka 1979; Hicks et al. 1994). Weather conditions during flowering can promote increased tree pollen concentrations in particular years (Spieksma et al. 2003). The occurrence of abundant pollen shed is also dependent on weather conditions in the summer of the previous year, since it is then that flower primordia are formed. Birch inflorescences start to develop in June of the previous year (Suszka 1979). There is a correlation between abundant pollen shed and the number of seeds produced in a given year. An abundant production of seeds occurs once every 2–3 years (Suszka 1979). Therefore, abundant birch pollen seasons can be expected every 2–3 years. In many sampling sites, the pollen monitoring programmes have found a biennial pattern of alternating high and low airborne birch pollen concentrations (Nilsson and Persson 1981; Ramfjord 1991; Spieksma et al. 1995; Latałowa et al. 2002), while in Iceland, a triennial cycle for annual pollen counts was observed (Hallsdóttir 1999). No clear biennial or triennial cycle of alternating high and low birch pollen concentrations was found in Lublin.

The climatic factor plays a major role in the production and release of pollen from anthers. Trends occurring in annual birch pollen counts are similar on a wider regional scale. Agreement in the trends in recorded pollen counts was found between sampling sites distant 120 km from each other and significantly differing in their surrounding vegetation (Pidek et al. 2006). There is high consistency in the rate of pollen release on the national scale, which is shown in a comparison of the features of the birch atmospheric pollen seasons in several Poland’s cities in the period 2001–2005. The same trends in annual pollen totals were observed in all the cities; the year 2001 was quite abundant, 2002 poor, 2003 very abundant, 2004 average, 2005 poor. The year 2003 clearly stood out from the other years in all the sampling sites (Weryszko-Chmielewska 2006). There was also a similar trend in annual pollen counts in Lublin and Riga in the period 2003–2008. High and low pollen concentrations were recorded in the same years. In 2003, an exceptionally high pollen concentration was observed in both cities (Pidek et al. 2009).

In spite of the fact that these trends of abundant and poor production of birch pollen are similar in various sampling sites, the values of annual pollen counts and the pattern of the atmospheric pollen seasons differ significantly (Weryszko-Chmielewska et al. 2001; Weryszko-Chmielewska 2006; Pidek et al. 2009). This is attributable to many factors that interact (among others, the plant cover of the immediate vicinity, topographic features of the area and anthropogenic factors). Among them, the most important are meteorological factors that are responsible for seasonal variability in the same sampling site in particular years.

In countries with temperate climate, air temperature recorded at the end of winter and in early spring has the greatest effect on the onset of the atmospheric pollen season. Birch pollen shed in eastern Poland starts on different days of April. A comparison of the start dates of the birch pollen season in 8 research centres in Poland shows that the season start dates vary much between years and between sites, and no region can be distinguished in which the pollen season would occur earlier each year than anywhere else (Weryszko-Chmielewska 2006).

The PCA analysis shows that the most favourable meteorological conditions (high temperature and low rainfall) during the pollen season were in the year 2003. This was the year in which exceptionally high pollen concentrations were recorded. The pollen season in 2006 was also warm, but more rainfall was recorded during that year than in 2003 (Fig. 2). The pollen seasons during which air temperature was at an average level belonged to the long seasons. Besides, low rainfall levels and the lowest pollen concentrations were observed during the longest seasons (2002 and 2009). Undoubtedly, meteorological conditions during the pollen season affect its pattern. However, further analysis showed that the birch pollen season characteristics depend mainly on the weather before the season.

Before flowering, trees need to accumulate a definite dose of thermal energy (so-called cumulative temperature; Suszka 1979; Frenguelli et al. 1992; Minero et al. 1999; Clot 2001). According to some authors, the thermal conditions during a period of 40 days before pollen shed have a decisive influence on the beginning of the pollen season; the higher cumulative temperature is above 5°C, the earlier the season starts (Spieksma et al. 1995; Latałowa et al. 2002; Myszkowska et al. 2007). In Lublin, this correlation was not statistically significant. Based on regression analysis, the thermal conditions in February were found to have the greatest effect on the birch atmospheric pollen season in Lublin. With lower temperature in February, the season was shorter and less intense. This relationship could also be seen in the earlier years of research carried out in Lublin (Piotrowska 2006). However, in the London area, the start of the birch pollen season depended on mean temperature of February and March. In regression analysis, an explanation at the level of 61.8% was obtained for these variables (Emberlin et al. 1993).

The duration of the birch atmospheric pollen season in Lublin shows large variability in particular years. Great variations in season duration have also been found in the studies carried out across Poland and Europe (Emberlin et al. 1993; Spieksma et al. 2003; Weryszko-Chmielewska and Piotrowska 2004; Weryszko-Chmielewska 2006). Our analysis shows that season duration is dependent primarily on minimum temperature of February and March, whereas according to Emberlin et al. (1993), it depends on rainfall and temperature at the end of the pollen season.

In the present study conducted in Lublin in the period 2001–2010, a relationship was shown between average birch pollen concentration during the season and minimum temperature of February. Pidek et al. (2009) also observed that the lower temperature in February was, the more abundant pollen shed was.

The long-term pollen monitoring data from many research centres show that there has been an increase in birch pollen concentrations over the recent years. Earlier bloom dates have also been observed. The global warming is considered to be the cause of this phenomenon (Emberlin et al. 1997; Frei 1998; Corden et al. 2000; Emberlin et al. 2002). Puc (2006) also found an increasing trend in annual birch pollen counts and pollen counts recorded in April. The longer pollen seasons and the higher pollen production, among others, are responsible for the increased incidence of pollinosis. Based on long-term research (42 years), it was found that the earlier start of the birch pollen season in many cities across Europe was associated with increased temperatures in the months from January to March (Spieksma et al. 2003). In the recent years, an increase in mean air temperature has also been observed in Lublin. In 2001–2010, the thermal conditions in the first months of the year (I-IV) were higher on average by 1°C than during the period 1951–2007; mean temperature in the successive months was follows: −2.2, −0.9, 3.1 and 9.7. In spite of this, no clear trend was found that would indicate the earlier onset of the birch atmospheric pollen season.

## Conclusions

The analysis of the meteorological data from the birch atmospheric pollen season demonstrates that pollen seasons were the warmest in 2003 and 2006, while in 2007 was the coldest one. Cloud cover, rainfall and high humidity were observed least frequently during APS in 2007 and 2009, whereas they were most frequent in the years 2005, 2008 and 2010. The most windless seasons were in 2001 and 2008.

In comparing the birch atmospheric pollen seasons in Lublin, the seasons in 2002, 2004, 2005 and 2009 were found to be similar, whereas the year 2003 differed the most. A distinct trend towards earlier occurrence of the date of the maximum birch pollen concentration was observed in the period 2001–2010.

The birch atmospheric pollen season is affected by weather both in the year before pollen release and in the same year in which flowering occurs. Statistical analysis of the correlations between meteorological factors and the start date of the birch pollen season in Lublin in the period 2001–2010 shows that minimum temperature of February had the greatest effect. Minimum temperature of February also influenced average pollen concentration during the season. Season duration was most correlated with minimum temperature of February and March, whereas the peak value was primarily dependent on rainfall in June in the year preceding pollen release. The obtained regression models account for 73–81% of the variations in the start date, season duration and the peak value. A poorer fit was obtained for average concentration of birch pollen during the season (64%). Based on the obtained results, it can be concluded that low temperatures in February promote the occurrence of high pollen concentrations. However, the obtained results need to be confirmed by long-term research.
